# Supplementary material for: Unravelling the role of vacancies in lead halide perovskite through electrical switching of photoluminescence
Source: Nat Commun. 2018 Nov 30;9:5113. doi: 10.1038/s41467-018-07571-6 (PMC6269531; doi:10.1038/s41467-018-07571-6)
Supplement: Supplementary file 1 — Supplementary Information [file 41467_2018_7571_MOESM1_ESM.docx]

**Supplementary Information:**

# Unravelling the role of vacancies in lead halide perovskite through electrical switching of photoluminescence

Cheng Li,^1^ Antonio Guerrero,^2^ Sven Huettner*,^1^ Juan Bisquert*^2^

^1^ Department of Chemistry, University of Bayreuth, Universitätstr. 30, 95447 Bayreuth, Germany.

^2^ Institute of Advanced Materials (INAM), Universitat Jaume I, 12006 Castello, Spain

Email: [sven.huettner@uni-bayreuth.de](mailto:sven.huettner@uni-bayreuth.de) [bisquert@uji.es](mailto:bisquert@uji.es)

This file includes:

Supplementary Figures 1 to 13

Supplementary Table 1

Supplementary Table 2

Supplementary Movie 1. Time dependent PL movie of a perovskite film CH_3_NH_3_PbI_3-x_Cl_x_ under an external electric field (approximately 2×10^4^ V m^-1^). The ‘+’ and ‘-‘ signs indicate the polarity of the electrodes. The excitation intensity is approximately 35 mW cm^-1^ with wavelength of 440 nm and the exposure time per image is 200 ms. The channel length is approximately 150 µm.

Supplementary Movie 2. Time dependent PL movie of a perovskite film CH_3_NH_3_PbI_3-x_Cl_x_ when applying positive and negative bias, subsequently. The ‘+’ and ‘-‘ signs indicate the polarity of the electrodes. The excitation intensity is approximately 35 mW cm^-1^ with wavelength of 440 nm and the exposure time per image is 200 ms. The channel length is approximately 150 µm.

Supplementary Figure 1. a) PL image of a perovskite film under an external electric field (approximately 2×10^4^ V m^-1^). *z(t)* represents the PL quenched area. The scale bar is 50 μm. b) The time dependent *z(t)* is shown. This time dependent curve is consistent with the measured time dependent current as well as the model.

Supplementary Figure 2. Time dependent current after a voltage of 4V has been applied. The initial short increase can be related to capacitive contributions after applying a voltage. These can be accounted for by an exponential saturation with a time constant of *RC* = 2.34 s. The Figure indicates that there exists a capacitive contribution due to the fast redistribution of ions at the beginning of the applied voltage. This explains the initial increase of current. Heiser, T. & Weber, E. R. Phys. Rev. B 58, 3893-3903 (1998) provides a detailed description of the transient ion drift induced capacitance signal.

Supplementary Figure 3. Time dependent PL images of a perovskite film under an external electrical field (approximately 1.3×10^5^ V m^-1^). The ‘+’ and ‘-‘ signs indicate the polarity of the electrodes. The excitation intensity is approximately 35 mWcm^-2^ and the exposure time per image is 200 ms. The channel length is approximately 150 µm. Within some of the samples, the PL active area cannot reach the opposite electrode, but the calculated mobility is still consistent with the other ones.

Supplementary Figure 4. Time dependent PL images of a 2D multiple quantum wells perovskite film (NFPI_7_) *(1)* under an external electrical field (approximately 3×10^5^ V m^-1^). The ‘+’ and ‘-‘ signs indicate the polarity of the electrodes. The excitation intensity is approximately 35 mW cm^-2^ and the exposure time per image is 200 ms. The scale bar is 100 µm.


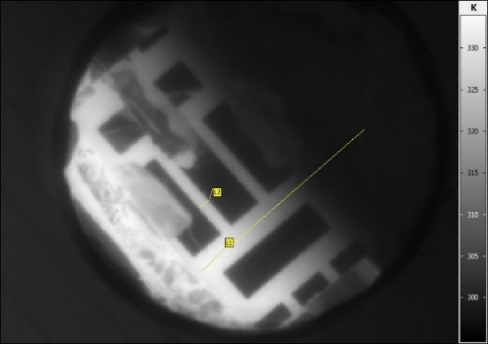


a)

b)

Electrode 1

Electrode 2

1mm gap

Supplementary Figure 5. a) Seebeck coefficient measurements of a MAPI_3-x_Cl_x_ layer as used in the experiment. The Seebeck coefficient was determined my measuring the thermal voltage which appears between 2 electrodes placed on a semiconductor within a temperature gradient.^3^ The temperature difference between a 1mm channel was adjusted by placing the sample between two blocks with two independently controlled temperatures. The slop increases from 3.4±0.5 mV K^-1^ to 6.2±0.8 mV K^-1^, implying a change of doping. b) An exact temperature calibration between the channel was performed with an IR-camera.

Supplementary Figure 6: Integrated and normalized PL intensity showing the PL profile within the channel. The quenched PL area is moving from left to right. The PL intensity increases through electroluminescence right at the border between region 1 and region 2.

**0**

**1000**

**2000**

**3000**

**4000**

**0**

**50**

**100**

**150**

**200**

**Intensity (a.u)**

**Distance**

**(**

**μ**

**m**

**)**


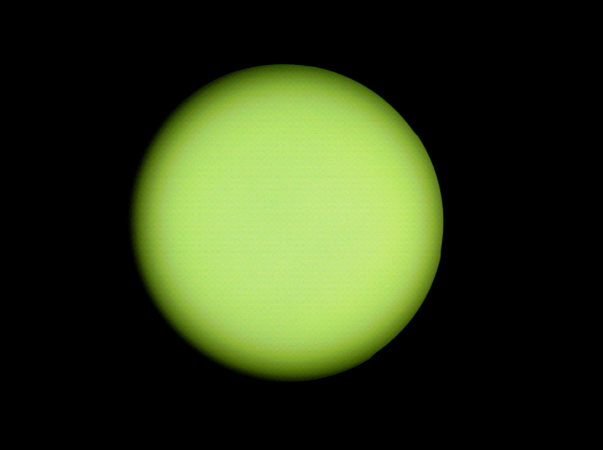


**b)**

**c)**

**a)**

Supplementary Figure 7. a) Schematic diagram for a PL imaging microscopy. b) Excited light beam (wavelength approximately 440 nm) image captured by a CCD camera. The scale bar is 1 mm. c) Intensity distribution of the excited beam in the focus plane. Based on this intensity profile, we can consider that at the central of the beam, it is a uniformly distributed light intensity, approximately 35 mW cm^-2^.

Supplementary Figure 8: Example of three representative fitting curves (solid lines) for MAPbI_x_Cl_3-x_ interdigitated electrodes using the proposed model.

|  |
| --- |

Supplementary Figure 9: Different responses on time dependent current before and after reversal of the polarity of the voltage.


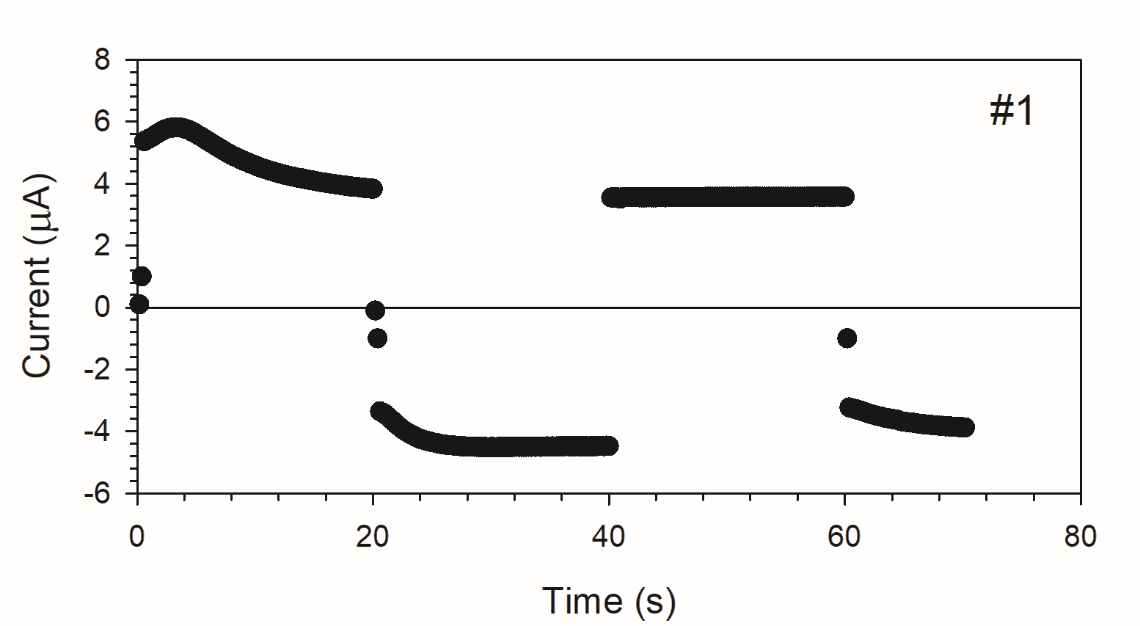


Supplementary Figure 10: Time dependent current during reversing the voltage 3 times.

Supplementary Figure 11. XPS survey spectrum of the perovskite film (MAPbI_x_Cl_3-x_), and the XPS spectra of Pb, I3d, and Cl2p, respectively. According to Fig d), there are a small amount of Cl element in the film.

Supplementary Figure 12. a) XRD and b) GIWAXS result of the perovskite film (MAPbI_x_Cl_3-x_). In Figure b), the white arrow indicates the small signal from intermediate phase.

Supplementary Figure 13. a) *J-V* curve of a MAPbI_x_Cl_3-x_ perovskite solar cell (FTO/TiO_2_/MAPbI_x_Cl_3-x_/Spiro-oMeTAD/Au) under AM 1.5 condition. b) *J-V* curve scanning at around *V_oc_*, with small voltage around 0.05V.

Supplementary Table 1: Calculated electrical field considering the device geometry and description on whether a dark front is observed.

|  | **Channel Length /** | | **Applied bias /**  ***V*** | **Electrical field /**  **V m^-1^** | **PL behaviour** |
| --- | --- | --- | --- | --- | --- |
|  | μm | m |  |  |  |
| PV Device | 0.35 | 3.5×10^-7^ | 0.2 | 5.7×10^5^ |  |
| PV Device | 0.35 | 3.5×10^-7^ | 0.5 | 1.4×10^6^ |  |
| PV Device | 0.35 | 3.5×10^-7^ | 1 | 2.9×10^6^ |  |
| Interdigitated | 150 | 1.5×10^-4^ | 0.2 | 1.3×10^3^ | General PL quenching, MAPbI_x_Cl_3-x_ |
| Interdigitated | 150 | 1.5×10^-4^ | 0.4 | 2.7×10^3^ | General PL quenching, MAPbI_x_Cl_3-x_ |
| Interdigitated | 150 | 1.5×10^-4^ | 1 | 6.7×10^3^ | Dark front, MAPbI_x_Cl_3-x_ |
| Interdigitated | 150 | 1.5×10^-4^ | 5 | 3.3×10^4^ | Dark front, MAPbI_x_Cl_3-x_ |
| Interdigitated | 150 | 1.5×10^-4^ | 10 | 6.7×10^4^ | Dark front, MAPbI_x_Cl_3-x_ |
| Interdigitated | 150 | 1.5×10^-4^ | 100 | 6.7×10^5^ | Dark front, MQW Perovskite |

Supplementary Table 2: Summary of calculations obtained for different samples

| Materials | Voltage / V | Slope / μA^-2^ s^-1^ | *j_0_*/  μA | *v_0_*/  μms^-1^ | *γ* | *μ*/  cm^2^V^-1^s^-1^ | *D*/  cm^2^s^-1^ |
| --- | --- | --- | --- | --- | --- | --- | --- |
| MAPbI_x_Cl_3-x_ | 5 | 2.6×10^-3^ | 5.7 | 11.36 | 0.6 | 2.15×10^-6^ | 5.60×10^-8^ |
| MAPbI_x_Cl_3-x_ | 7.5 | 1.1×10^-2^ | 10.2 | 22 | 4.1 | 8.53×10^-7^ | 2.21×10^-8^ |
| MAPbI_x_Cl_3-x_ | 10 | 6.0×10^-3^ | 5.1 | 5 | 2.3 | 2.25×10^-7^ | 6.00×10^-9^ |
| MAPbI_x_Cl_3-x_ | 10 | 2.4×10^-5^ | 41 | 41 | 1.42 | 2.54×10^-6^ | 6.06×10^-8^ |
| (FA_0.83_MA_0.17_)_0.95_Cs_0.05_Pb(I_0.9_Br_0.1_)_3_ | 10 | 5.9×10^-4^ | 4.3 | 3.09 | 0.23 | 3.27×10^-7^ | 8.47×10^-9^ |
| Perovskite MQW^[1]^ | 100 | 1.7×10^3^ | 2.2×10^-2^ | 0.65 | 0.53 | 8.5×10^-9^ | 2.20×10^-10^ |

**Supplementary References:**

1. N. Wang *et al.,* Perovskite light-emitting diodes based on solution-processed self-organized multiple quantum wells, *Nat. Photon.***10**, 699 (2016).

2. C. Li *et al.*, Real-Time Observation of Iodide Ion Migration in Methylammonium Lead Halide Perovskites. *Small* ***13,*** 1701711 (2017).

3. J. Ravichandran *et al*., An apparatus for simultaneous measurement of electrical conductivity and thermopower of thin films in the temperature range of 300–750 K, *Rev. Sci. Instrum.* **82**, 015108 (2011).
