# Supplementary material for: Unravelling the role of vacancies in lead halide perovskite through electrical switching of photoluminescence
Source: Nat Commun. 2018 Nov 30;9:5113. doi: 10.1038/s41467-018-07571-6 (PMC6269531; doi:10.1038/s41467-018-07571-6)
Supplement: Supplementary file 3 — Description of Additional Supplementary Files [file 41467_2018_7571_MOESM3_ESM.pdf]

Description of Additional Supplementary

Supplementary Movie 1. Time dependent PL movie of a perovskite film $CH_3NH_3PbI_{3-x}Cl_x$ under an external electric field (approximately 2×10^4 V m^{-1}). The '+' and '-' signs indicate the polarity of the electrodes. The excitation intensity is approximately 35 mW cm^{-1} with wavelength of 440 nm and the exposure time per image is 200 ms. The channel length is approximately 150 µm.

Supplementary Movie 2. Time dependent PL movie of a perovskite film $CH_3NH_3PbI_{3-x}Cl_x$ when applying positive and negative bias, subsequently. The '+' and '-' signs indicate the polarity of the electrodes. The excitation intensity is approximately 35 mW cm^{-1} with wavelength of 440 nm and the exposure time per image is 200 ms. The channel length is approximately 150 µm.
